# Biomarkers Predict In-Hospital Major Adverse Cardiac Events in COVID-19 Patients: A Multicenter International Study

**DOI:** 10.3390/jcm10245863

**Published:** 2021-12-14

**Authors:** Michael Y. Henein, Giulia Elena Mandoli, Maria Concetta Pastore, Nicolò Ghionzoli, Fouhad Hasson, Muhammad K. Nisar, Mohammed Islam, Francesco Bandera, Massimiliano M. Marrocco-Trischitta, Irene Baroni, Alessandro Malagoli, Luca Rossi, Andrea Biagi, Rodolfo Citro, Michele Ciccarelli, Angelo Silverio, Giulia Biagioni, Joseph A. Moutiris, Federico Vancheri, Giovanni Mazzola, Giulio Geraci, Liza Thomas, Mikhail Altman, John Pernow, Mona Ahmed, Ciro Santoro, Roberta Esposito, Guillem Casas, Rubén Fernández-Galera, Maribel Gonzalez, Jose Rodriguez Palomares, Ibadete Bytyçi, Frank Lloyd Dini, Paolo Cameli, Federico Franchi, Gani Bajraktari, Luigi Paolo Badano, Matteo Cameli

**Affiliations:** 1 Department of Public Health and Clinical Medicine, Umeå University, 90187 Umeå, Sweden; i.bytyci@hotmail.com (I.B.); franklloyddini@gmail.com (F.L.D.); ganibajraktari@yahoo.co.uk (G.B.); 2 St George London and Brunel Universities, London SW17 0QT, UK; 3 Department of Medical Biotechnologies, Division of Cardiology, University of Siena, 53100 Siena, Italy; giulia_elena@hotmail.it (G.E.M.); nicologhionzoli@gmail.com (N.G.); giuliabiagioni13@gmail.com (G.B.); matteo.cameli@yahoo.com (M.C.); 4 Luton and Dunstable University Hospital, NHS Foundation Trust, Luton LU4 0DZ, UK; fouad.hasson@ldh.nhs.uk (F.H.); muhammad.nisar@ldh.nhs.uk (M.K.N.); mohammed.islam2@ldh.nhs.uk (M.I.); 5 Department for Biomedical Sciences for Health, University of Milano, 20133 Milan, Italy; francescobandera@gmail.com; 6 Cardiology University Department, Heart Failure Unit, IRCCS Policlinico San Donato, San Donato Milanese, 20097 Milan, Italy; 7 Clinical Research Unit, IRCCS Policlinico San Donato, San Donato Milanese, 20097 Milan, Italy; trischitta@gmail.it (M.M.M.-T.); irebaroni@gmail.it (I.B.); 8 Division of Cardiology, Nephro-Cardiovascular Department, Baggiovara Hospital, University of Modena and Reggio Emilia, 41126 Modena, Italy; ale.malagoli@gmail.com; 9 Division of Cardiology, Cardiovascular and Emergency Department, Guglielmo da Saliceto Hospital, 29121 Piacenza, Italy; dr.luca.rossi.20@gmail.com (L.R.); a.biagi88@gmail.com (A.B.); 10 Cardio-Thoracic-Vascular Department, University Hospital San Giovanni di Dio e Ruggi d’Aragona, 84125 Salerno, Italy; rodolfocitro@gmail.com; 11 Department of Medicine, Surgery and Dentistry, University of Salerno, 84081 Baronissi, Italy; mciccarelli@unisa.it (M.C.); asilverio@unisa.it (A.S.); 12 Paphos Hospital, University of Nicosia, Nicosia 8100, Cyprus; moutiris@hotmail.com; 13 Department of Internal Medicine, S. Elia Hospital, 93100 Caltanissetta, Italy; fvanche@tin.it (F.V.); giomazzola@gmail.it (G.M.); giugeraci@gmail.it (G.G.); 14 Department of Cardiology, Westmead Hospital and Westmeead Clinical School, University of Sydney, Sydney, NSW 2145, Australia; liza.thomas@sydney.edu.au (L.T.); mikaltman@gmail.it (M.A.); 15 Department of Medicine, Division of Cardiology, Karolinska Institutet, 17177 Stockholm, Sweden; John.Pernow@ki.se; 16 Department of Molecular Medicine and Surgery, Division of Cardiology, Karolinska Institutet, 17177 Stockholm, Sweden; mona.ahmed@ki.se; 17 Department of Advanced Biomedical Sciences, University of Naples “Federico II”, 80131 Naples, Italy; cirohsantoro@gmail.com; 18 Department of Clinical Medicine and Surgery, Federico II University Hospital, 80131 Naples, Italy; robertaesposito6@gmail.com; 19 Department of Cardiology, Hospital Universitari Vall d’Hebron, Vall d’Hebron Institut de Recerca (VHIR), Universitat Autònoma de Barcelona, 08035 Barcelona, Spain; gcasasmasnou@gmail.com (G.C.); rubenfdezgalera@gmail.com (R.F.-G.); marigonzalez@gmail.it (M.G.); jrpalomares@gmail.it (J.R.P.); 20 Clinic of Cardiology, University Clinical Centre of Kosova, 10000 Prishtina, Kosovo; 21 Respiratory Diseases Unit, Department of Medical Sciences, Siena University Hospital, 53100 Siena, Italy; paolocameli88@gmail.com; 22 Department of Medical Biotechnologies, Anesthesia and Intensive Care, University of Siena, 53100 Siena, Italy; federico.franchi@unisi.it; 23 Medical Faculty, University of Prishtina “Hasan Prishtina”, 10000 Prishtina, Kosovo; 24 Istituto Auxologico Italiano, IRCCS, 20149 Milan, Italy; lpbadano@gmail.com; 25 Department of Medicine and Surgery, University of Milano-Bicocca, 20126 Milan, Italy

**Keywords:** COVID-19, SARS-CoV2, biomarkers, troponin, creatinine, prognosis

## Abstract

Background: The COVID-19 pandemic carries a high burden of morbidity and mortality worldwide. We aimed to identify possible predictors of in-hospital major cardiovascular (CV) events in COVID-19. Methods: We retrospectively included patients hospitalized for COVID-19 from 10 centers. Clinical, biochemical, electrocardiographic, and imaging data at admission and medications were collected. Primary endpoint was a composite of in-hospital CV death, acute heart failure (AHF), acute myocarditis, arrhythmias, acute coronary syndromes (ACS), cardiocirculatory arrest, and pulmonary embolism (PE). Results: Of the 748 patients included, 141(19%) reached the set endpoint: 49 (7%) CV death, 15 (2%) acute myocarditis, 32 (4%) sustained-supraventricular or ventricular arrhythmias, 14 (2%) cardiocirculatory arrest, 8 (1%) ACS, 41 (5%) AHF, and 39 (5%) PE. Patients with CV events had higher age, body temperature, creatinine, high-sensitivity troponin, white blood cells, and platelet counts at admission and were more likely to have systemic hypertension, renal failure (creatinine ≥ 1.25 mg/dL), chronic obstructive pulmonary disease, atrial fibrillation, and cardiomyopathy. On univariate and multivariate analysis, troponin and renal failure were associated with the composite endpoint. Kaplan–Meier analysis showed a clear divergence of in-hospital composite event-free survival stratified according to median troponin value and the presence of renal failure (Log rank *p* < 0.001). Conclusions: Our findings, derived from a multicenter data collection study, suggest the routine use of biomarkers, such as cardiac troponin and serum creatinine, for in-hospital prediction of CV events in patients with COVID-19.

## 1. Introduction

During the last two years, the coronavirus disease 2019 (COVID-19) pandemic affected millions, and it is currently afflicting national health services worldwide, with its high morbidity and mortality in severely ill patients, with more than 5 million deaths [1]. COVID-19 disease is caused by severe acute respiratory syndrome coronavirus 2 (SARS-CoV2) infection, which leads to a wide spectrum of clinical manifestations, ranging from complete lack of symptoms to aggressive interstitial bilateral pneumonia with a high fatality rate.

The pathophysiology of COVID-19 comprises initial respiratory tract infection, followed by an exaggerated inflammatory response, caused by the viral infection, leading to a prothrombotic, procoagulant, and endothelial dysfunction with potential multi-organ involvement [2,3]. Cardiac injury can also be produced through direct mechanisms involving viral infiltration into the myocardial tissue, resulting in cardiomyocyte lysis and inflammation, and indirect mechanisms including cardiac stress due to respiratory failure and hypoxemia or cardiac inflammation secondary to an aggressive systemic inflammatory process. Such cardiac involvement leads to raised biomarker (cardiac troponin I and brain-type natriuretic peptide) levels, arrhythmias, myocardial infarction, and heart failure [4,5,6].

The scientific community has and is actively evaluating various aspects of COVID-19 disease in order to improve diagnosis and treatment [7,8,9]; however, the prognostic evaluation of poor in-hospital outcome in these patients still remains challenging [10]. Pre-existing cardiovascular risk factors and many biomarkers, among which cardiac and renal biomarkers, have been shown to correlate with severe complications of COVID-19 [11,12,13,14], although not necessarily with cardiovascular events.

The aim of this multicenter international study, involving 10 centers in five countries in Europe and Australia (Italy, Sweden, United Kingdom, Australia (Sydney), Spain), was to determine possible predictors of in-hospital major adverse cardiac events in a large cohort of patients hospitalized for COVID-19.

## 2. Materials and Methods

### 2.1. Study Design

In this retrospective study, we collected data from 10 COVID-19 specialized referral centers, mainly European (See Table A1 for the list of all centers). All patients were admitted between January and October 2020 with SARS-CoV2 infection. They required hospitalization and were all ≥18 years of age at the time of admission. The diagnosis of COVID-19 infection was determined by positive oro-/naso-pharyngeal real-time PCR swab test for SARS-CoV2 nucleic acid along with respiratory and/or gastrointestinal symptoms. Countries involved were, listed in alphabetical order: Australia, Italy, Spain, Sweden, United Kingdom. Each center contributed with at least 20 patients.

Baseline characteristics were obtained at admission for patients who could communicate and were collected from general practitioners and families by independent investigators in patients who could not communicate. Vital signs assessed at admission were collected from electronic medical records and emergency department files, as appropriate. In particular, fever was defined as intra-auricular temperature >38 °C, hypotension as systolic artery pressure ≤ 100 mmHg, tachycardia as heart rate ≥100 beats per minute, and hypoxemia as peripheral finger oxygen saturation ≤92%. All patients had undergone a thorough physical examination at admission, and blood samples were sent for a series of hematological and biochemical investigations including, hemoglobin, white blood cells, platelets, C reactive protein (CRP), serum creatinine, electrolytes, indices of hepatic function, and high-sensitivity cardiac troponin. All centers used the same laboratory methods for biomarkers assessment. Major electrocardiographic abnormalities were recorded. Echocardiography was performed based on clinical need, following the published recommendations on the optimization of echocardiographic examinations during the COVID-19 pandemic [15,16]. All echocardiographic measurements were made according to the recommendations of the American Society of Echocardiography (ASE)/European Association of Cardiovascular Imaging (EACVI) [17]. Medical treatment at admission, during hospitalization, and at discharge was documented for each patient. Other imaging and functional tests were performed, as clinically indicated. Patients were divided into two groups according to the development of a major adverse endpoint of combined cardiovascular events: patients who met the endpoint, and patients who did not.

This study was approved by the Swedish Ethics Board (Dnr 2020-02217 Stockholm avdelning 2 medicin) and also by local ethics committees in each site involved. An informed consent for the anonymous use of data was obtained directly by patients or by legal guardians. All study procedures complied with the Declaration of Helsinki.

### 2.2. Study Outcomes

Patients were followed up for any in-hospital clinical event, including all-cause and cardiovascular mortality. In-patient deaths were recorded according to standard procedures. A composite of adverse in-hospital cardiovascular events, including cardiovascular death (i.e., death resulting from acute myocardial infarction, sudden cardiac death, acute heart failure, acute pulmonary embolism, stroke), acute coronary syndromes (defined as acute chest pain or chest pain-equivalent or hemodynamic instability with/without troponin rise and the presence of obstructive coronary lesions on coronary angiography), acute heart failure, pulmonary embolisms, cardio-circulatory arrest, acute myocarditis (confirmed by cardiac magnetic resonance imaging according to the Lake Louise criteria [18] after exclusion of obstructive coronary disease on invasive coronary angiography), and life-threatening arrhythmias (i.e., sustained ventricular tachycardia, ventricular fibrillation, torsade-de-pointes, pulseless electrical activity), was created to represent the composite primary endpoint of the study. For acute coronary syndromes, raised high-sensitivity cardiac troponin T value above the upper reference limit (set to >22 ng/L for males and >14 ng/L for females) was considered significant. If multiple events were registered for a patient, it was only accounted as a single endpoint.

### 2.3. Statistical Analysis

Statistical analysis was performed using IBM SPSS 25.0 program (1989–2017, LEAD Technologies Inc., Armonik, NY, USA). The type of distribution of variables was assessed using the Kolmogorov–Smirnov test. Continuous variables with normal distribution are presented as mean ± standard deviation, and continuous variables with non-normal distribution as median and interquartile range. Non-continuous variables are presented as frequency and percentage.

Differences between groups (patients who met the composite primary endpoint and those who did not) were assessed using the Student *t*-test for independent samples or Mann–Whitney U test as appropriate for continuous variables. To assess differences in non-continuous variables, chi-square test or Fisher exact probability test were used, as appropriate. In each of these tests, two tailed *p* values < 0.05 were considered significant.

Univariate Cox regression analysis was performed in order to identify potential predictors of the composite endpoint. A multivariate model of Cox analysis was then built. Covariates were selected a priori based on clinical relevance and statistically significant differences between those who did and did not suffer the composite end point. The parameters that emerged on multivariate analysis as associated with the composite primary outcome were used to perform event-free survival analysis. Subsequently, the composite of in-hospital cardiovascular events was assessed by Kaplan–Meier analysis, after classifying the population according to the presence of renal failure (defined as creatinine ≥1.25 mg/dL, as obtained by receiver operating curves for the composite outcome) and above/below high-sensitivity troponin T median values. The Kaplan–Meier curve is presented at 60 days. Differences between groups were tested with the log-rank test (Mantel–Cox).

## 3. Results

Our cohort was composed of 748 patients, collected from 10 centers from different European countries and Australia. All patients were diagnosed with SARS-CoV2 disease and required hospital admission. Overall study population features are presented in Table 1.

### 3.1. Follow-Up

**Figure 1:**
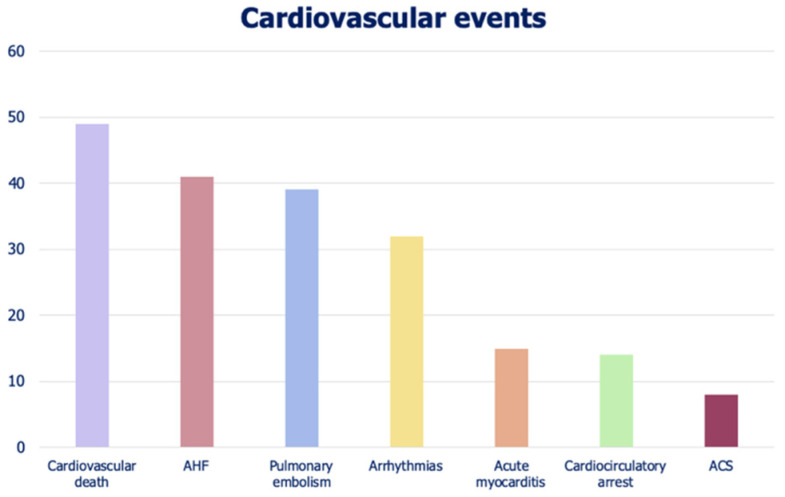
Numerical distribution of cardiovascular events in our study cohort.

Over a mean follow-up of 18 days, 141 patients (19%) reached the combined primary endpoint. The total number of CV events was 198, as 57 patients experienced more than one event. Events were classified as: 49 (7%) CV deaths, 15 (2%) acute myocarditis, 32 (4%) sustained-supraventricular or ventricular arrhythmias, 14 (2%) cardiocirculatory arrests, 8 (1%) acute coronary syndromes, 41 (5%) acute heart failure, and 39 (5%) pulmonary embolisms. The distribution of CV events in the study cohort is shown in Figure 1. The in-hospital follow-up was significantly longer in patients with CV events due to longer hospitalization (*p* = 0.041).

The all-cause mortality of the whole cohort was 211 patients (28%), with CV deaths accounting for 22% of them. Cardiovascular events, whether fatal or not, were detected in 141 (19%) patients.

We compared the two sub-populations, based on those who experienced the composite primary end point (i.e., CV events, see Methods). A descriptive analysis of baseline clinical characteristics, laboratory and electrocardiographic findings, and therapy at admission, in the two groups, is shown in Table 1.

### 3.2. Clinical and Biohumoral Characteristics

Compared with patients without CV events at follow-up, those with events were older (*p* = 0.008) and with a higher CV risk profile: more prevalent systemic hypertension (*p* = 0.008) and renal failure (*p* = 0.024). Patients with CV events also had more comorbidities including, atrial fibrillation (AF) (*p* = 0.013), an underlying cardiomyopathy (*p* = 0.036), and/or chronic obstructive pulmonary disease (COPD) (*p* = 0.013). Finally, patients with CV events had higher body temperature at presentation (*p* = 0.042), whereas the other vital signs, including peripheral blood oxygen saturation, were not different between groups.

**Figure 2:**
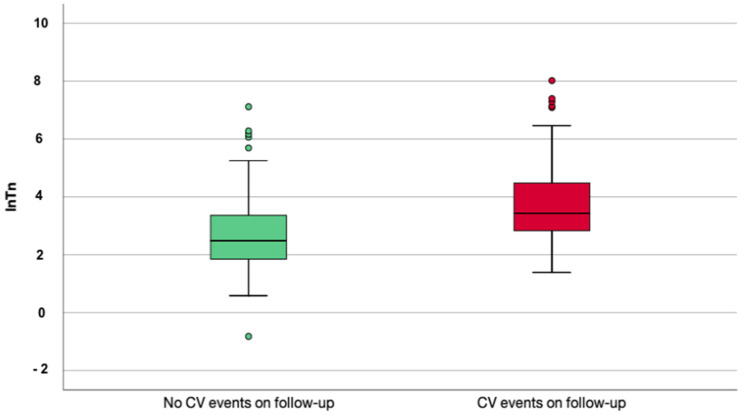
Box plots describing troponin values in patients who reached the composite outcome and who did not reach the composite outcome.

Again, in-patients with CV events blood results showed higher total white blood cells (*p* < 0.001) and platelets count (*p* = 0.007) and higher high-sensitivity troponin level (*p* < 0.001) at admission. Differences in median values of troponin are shown in Figure 2. Electrocardiographic parameters, such as QRS and PR durations and QT interval, were not different between the two groups of patients.

Patients with CV events were more frequently treated with angiotensin-converting enzyme inhibitors/angiotensin receptor antagonists (ACEi/ARB) (*p* = 0.046) and diuretics (*p* = 0.001) at admission, and they more frequently received anticoagulants during hospitalization (*p* = 0.001). They were also more frequently treated with non-invasive artificial ventilation (27% vs. 16%; *p* = 0.009).

### 3.3. Biomarkers Associated with In-Hospital Cardiovascular Events

On univariate analysis, age, renal failure, COPD and AF, and lower levels of peripheral oxygen saturation and troponin levels were associated with CV events. In contrast, medical treatment with diuretics and anticoagulants were associated with a lower incidence of such events (*p* = 0.003 and *p* = 0.002, respectively). Hypertension, body temperature, white blood cells count, platelets count, sodium levels, and use of ACEi/ARB were not significantly associated with CV events on univariate analysis. When a multivariate model was created, only renal failure and troponin levels at baseline were independently associated with the occurrence of CV events (*p* = 0.005 and *p* = 0.003, respectively) (Table 2).

**Figure 3:**
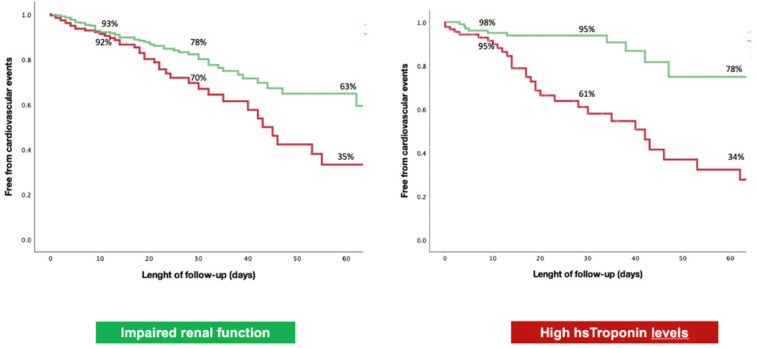
Kaplan–Meier curves for the prediction of the composite outcome in our study cohort stratified for presence/absence of renal failure at baseline (**left**) and for median troponin value (**right**).

Kaplan–Meier analyses by presence/absence of renal failure and median troponin value for CV events are presented in Figure 3. When evaluating the population based on the presence of renal failure, curves tended to diverge at 10 to 15 days, with significance on Mantel–Cox (Log rank *p* = 0.011). When identifying cohorts with troponin values above and below the median value, curves tended to diverge at about 10 days (Log rank *p* < 0.001).

## 4. Discussion

Our multicenter international study, involving 10 centers and five countries worldwide, identified baseline cardiac troponin and creatinine at admission to be associated with in-hospital adverse cardiovascular events, including CV death, acute coronary syndromes, acute heart failure, pulmonary embolisms, cardiocirculatory arrest, acute myocarditis, and life-threatening arrhythmias.

These results come as a confirmation and validation of previous evidence (6–8; 18–20) from multicenter studies. A systematic review including 34 studies showed that cardiac troponin, renal biomarkers (creatinine, estimated glomerular filtration rate (eGFR), blood urea), together with C-reactive protein, serum amyloid A, interleukin-6, lactate dehydrogenase, neutrophil-to-lymphocyte ratio, D-dimer, lymphocytes, and platelet count were associated with COVID-19 severity [13]. However, the authors themselves acknowledged many limitations of their results, including that data were derived from single-center studies from Wuhan, with possible inclusion of the same patients in different studies, and they stated the need for further data collection worldwide and advanced statistical analysis to confirm their initial results.

### 4.1. Renal Disease in COVID-19

Many studies analyzed the relation between pre-existing renal disease and worse outcome in the form of higher mortality [19,20,21], need for hospitalization, and intensive care admission in patients with SARS-CoV2 infection [22]. A propensity-score match study conducted in a multicenter study of electronic medical records in a cohort of 152,463 patients found that any grade and in particular moderate chronic kidney disease (CKD) was an independent risk factor for more severe COVID-19 disease presentation and mortality, after accounting for other coexistent comorbidities [23]. This suggests the need for early recognition of patients with CKD as a high-risk population among those with COVID-19. Interestingly, Li et al. studied the impact of acute cardiac and renal injury in patients hospitalized for COVID-19 and found that such patients had a considerably higher severity-rate and mortality-rate when compared with those without acute cardiac or kidney injury [24].

### 4.2. The Use of Cardiac Troponin in COVID-19

The incidence of acute direct or indirect cardiac injury in COVID-19 [5] results in a raised cardiac troponin that could be related to mortality [25]. This finding developed a high scientific interest in utilizing cardiac biomarkers as diagnostic and prognostic tools for long-term cardiac complications related to SARS-CoV2 infection. Cardiac troponin has already been shown to be a good prognosticator in patients with COVID-19, being a predictor of mortality, intensive care unit admission, and myocardial injury [26,27,28,29,30,31,32]. This was confirmed in a meta-analysis involving 10 studies with a total of 3982 patients, in which higher troponin levels carried a considerable higher risk of admission to intensive care units, oxygen saturation <90%, invasive mechanical ventilation, and in-hospital mortality (odds ratio = 7.92, 95% confidence interval: 3.70–16.97; *p* < 0.00001). On the other hand, Al Abbasi et al. found a high negative predictive value of a normal troponin-I level in the first 24 h of admission in predicting in-hospital mortality from any cause [33]. In this setting, the use of echocardiography could be pivotal for early recognition of primary or COVID-19-induced myocardial damage [34]; however, it should be reserved for only cases in doubt, as an important precaution for limiting the risk of contagion. This makes the use of biomarkers even more important for diagnostic and prognostic purposes [35].

Our results add important information to the current evidence in confirming the utility of cardiac troponin as the main parameter, associated beyond CV mortality, with other potentially life-threatening complications or acute coronary syndrome. This strengthens the importance of considering troponin as one of the main parameters to be routinely assessed in these patients for an early prediction of in-hospital outcome. Thus, systematic measurement of cardiac troponin may optimize risk stratification and may aid in better disease categorization and phenotyping among patients hospitalized for COVID-19.

### 4.3. Limitations

Even though the results of our study are promising, being derived from multicenter standardized data collection, some limitation should be acknowledged. First, the small sample size and the retrospective nature of the study, which led to limited and missing data, hence the narrow scale analysis that was performed. We were not able to account for body weight and body mass index in the multivariate analysis due to missing data in hospital records. Moreover, the use of estimated glomerular filtration rate to define renal function was not available in all patients, depending on center’s clinical policy rather than the currently available universal protocols. Acute myocarditis was diagnosed by cardiac magnetic resonance, but confirmatory data by endomyocardial biopsy were not available. Echocardiographic and other imaging data were available for only one-third of the patients, since the EACVI recommendation on the optimization of echocardiography was not strictly followed, particularly in the first wave of the pandemic, when the current study data were collected.

## 5. Conclusions

The challenging management of COVID-19 patients has led to the timely need for reliable and commonly available prognostic markers to identify patients who are more likely to develop severe complications. Biomarkers are quantitative measurements that could be easily used to reflect higher risk and poorer clinical outcome in COVID-19 patients admitted to hospital. Our findings, derived from a multicenter data collection, suggest the use of cardiac troponin and serum creatinine to predict the development of possible in-hospital major adverse cardiovascular events in patients suffering from SARS-CoV2.

## Figures and Tables

**Table 1:** Baseline clinical features, laboratory and electrocardiographic findings, and therapy at admission of our study cohort.

	Whole Population (n = 748)	No CV Events (n = 607)	CV Events (n = 141)	*p*-Value
**Baseline Features**				
Age (years)	67 ± 16	65 ± 17	72 ± 15	**0.008**
Sex (female)	274 (37)	226 (37)	48 (34)	0.604
Hypertension n, (%)	402 (54)	312 (50)	90 (64)	**0.008**
Diabetes	198 (26)	155 (24)	43 (30)	0.187
Dyslipidemia	155 (20)	124 (19)	31 (21)	0.704
Renal failure	243 (30)	184 (28)	59 (39)	**0.024**
Chronic obstructive pulmonary disease	105 (13)	77 (11)	28 (19)	**0.013**
History of AF	81 (11)	57 (9)	24 (17)	**0.013**
Systolic blood pressure (mmHg)	127 ± 22	127 ± 21	128 ± 22	0.385
Heart rate (bpm)	87 ± 18	87 ± 17	128 ± 22	0.849
Temperature (°C)	37.4 ± 1.0	37.4 ± 1.0	37.6 ± 1.1	**0.042**
Saturation (%)	84 ± 29	84 ± 29	84 ± 28	0.942
Length of follow up (days)	18 ± 17	18 ± 16	21 ± 20	**0.041**
**Laboratory findings**				
Hemoglobin (g/dL)	13.0 ± 2.1	13.0 ± 2.1	13.0 ± 2.3	0.133
White blood cells (cells/mmc)	1234 ± 3286	1085 ± 2837	1990 ± 4934	**<0.001**
Platelets (cells/mmc)	228408 ± 102893	226385 ± 98926	239968 ± 123121	**0.007**
C reactive protein (mg/dL)	15.10 (5.60–53.50)	16.00 (6.02–56.00)	11.84 (4.75–38.83)	0.713
Serum creatinine (mg/dL)	1.16 ± 0.91	1.14 ± 0.93	2 ± 0.83	**<0.001**
Sodium (mEq/L)	138 ± 6	138 ± 5	138 ± 7	**0.24**
Potassium (mEq/L)	4.1 ± 0.5	4.1 ± 0.5	4.0 ± 0.1	0.146
Troponin (ng/L)	16 (7–40)	12 (6–29)	31 (17–94)	**<0.001**
ALT (IU/L)	35 ± 34	37 ± 35	30 ± 23	0.225
**Electrocardiographic findings**				
Supraventricular arrhythmias	91 (12)	69 (11)	22 (16)	0.214
**Therapy**				
ACE inhibitors/ARB	252 (34)	192 (32)	60 (42)	**0.046**
Beta blockers	153 (21)	122 (20)	31 (22)	0.701
MRA	24 (3)	21 (4)	3 (2)	0.587
CCB	110 (15)	84 (14)	26 (18)	0.208
Diuretics	115 (15)	80 (13)	25 (18)	**0.001**
Antiarrhythmics	48 (6)	39 (7)	9 (6)	0.418
Antiplatelet drugs	110 (15)	84 (14)	26 (18)	0.208
Anticoagulants	71 (10)	47 (8)	24 (17)	**0.001**
Corticosteroids	67 (9)	51 (8)	16 (11)	0.427

ACE, angiotensin converting enzyme; AF, atrial fibrillation; ALT, alanine aminotransferase; ARB, angiotensin receptor blocker; CCB, calcium channel blocker; MRA, mineralcorticoid receptor antagonist. Numbers in bold show statistical significance.

**Table 2:** Univariate and multivariate analysis of selected variables for the prediction of the primary composite outcome.

	Unadjusted HR (CI (95%))	Unadjusted *p*-Value	Adjusted HR (CI (95%))	Adjusted *p*-Value
Age	1.025 [1.012–1.038]	<0.001	0.991 [0.968–1.016]	0.481
Renal failure	1.605 [1.407–1.890]	0.013	1.314 [1.139–1.706]	**0.005**
Chronic obstructive disease	0.628 [0.401–0.982]	0.041	0.746 [0.275–2.021]	0.621
Oxygen saturation	0.992 [0.985–0.999]	0.027	0.998 [0.976–1.021]	0.805
History of AF	0.579 [0.371–0.902]	0.016	4.737 [0.314–7.134]	0.311
Troponin	1.607 [1.346–1.918]	<0.001	1.396 [1.122–1.737]	**0.003**
Diuretics	0.55 [0.372–0.813]	0.003	0.523 [0.194–1.413]	0.201
Anticoagulants	0.490 [0.314–0.764]	0.002	0.489 [0.031–7.741]	0.611

AF, atrial fibrillation; CI, confidence interval; HR, hazard ratio. Numbers in bold show statistical significance.

## Data Availability

The data presented in this study are available on request from the corresponding author.

## Appendix A

**Table A1:** Centers involved in the recruitment phase of our study cohort.

Luton Hospital, United Kingdom	213 patients
San Donato Hospital, Milan, Italy	200 patients
Guglielmo da Saliceto Hospital, Piacenza, Italy	78 patients
San Giovanni di Dio e Ruggi d’Aragona University Hospital, Salerno, Italy	73 patients
Santa Maria alle Scotte University Hospital, Siena, Italy	71 patients
S.Elia Hospital, Caltanissetta, Italy	27 patients
Westmead Hospital, Sydney, Australia	25 patients
Karolinska Institute, Stockholm, Sweden	21 patients
Federico II University Hospital, Naples Italy	20 patients
Hospital Universitari Vall d’Hebron, Barcelona, Spain	20 patients
